# Folic Acid Antimetabolites (Antifolates): A Brief Review on Synthetic Strategies and Application Opportunities

**DOI:** 10.3390/molecules27196229

**Published:** 2022-09-22

**Authors:** Igor S. Kovalev, Grigory V. Zyryanov, Sougata Santra, Adinath Majee, Mikhail V. Varaksin, Valery N. Charushin

**Affiliations:** 1 Department of Organic & Biomolecular Chemistry, Ural Federal University, 620002 Yekaterinburg, Russia; 2 I. Ya. Postovskiy Institute of Organic Synthesis, Ural Branch, Russian Academy of Sciences, 620219 Yekaterinburg, Russia; 3 Department of Chemistry, Visva-Bharati (A Central University), Santiniketan 731235, India

**Keywords:** antifolates, folic acids, synthesis, applications, drugs

## Abstract

Antimetabolites of folic acid represent a large group of drugs and drug candidates, including those for cancer chemotherapy. In this current review, the most common methods and approaches are presented for the synthesis of therapeutically significant antimetabolites of folic acid, which are Methotrexate (MTX), Raltitrexed (Tomudex, ZD1694), Pralatrexate, Pemetrexed, TNP-351, and Lometrexol. In addition, the applications or uses of these folic acid antimetabolites are also discussed.

## 1. Introduction

Antimetabolites, which are antagonists of natural metabolites, belong to a group of highly efficient anticancer drugs. Based on the chemical structure, these groups can be divided into several sub-groups, such as non-natural amino-acids [1] or peptides [2,3], including phospha-analogues [4], analogues of purine and pyrimidine bases, such as competitors in the synthesis of the nucleic acids [5,6], as well as vitamin actions including folic acid [7], hormones [8], coenzymes [9], and other substrates responsible for the normal functioning of cells and tissues of the human body.

The mechanism of antimetabolites action is based on their ability to enter into competitive relationships with structurally similar metabolites of the living, which leads to a lack of the corresponding metabolite and a decrease in the activity of vital biochemical processes in the cell. In order to interfere with the synthesis of the DNA constituents, the most common antimetabolites should be structural analogues of purine and pyrimidine bases/nucleosides, or of folate cofactors [10].

In this current review, we analyze the most common approaches for the synthesis of therapeutically significant antimetabolites of folic acid [11,12,13], such as Methotrexate (MTX), Raltitrexed (Tomudex, ZD1694), Pralatrexate, Pemetrexed, TNP-351, and Lometrexol.

## 2. Discussion

### 2.1. Mechanism of Antifolates Action

**Scheme 1:**
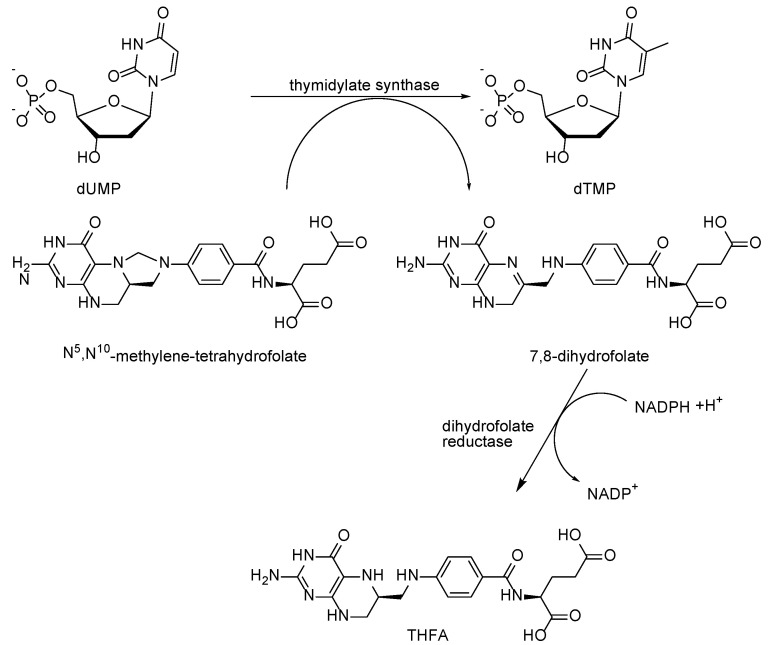
Main target processes for antifolates in living cells.

**Figure 1:**
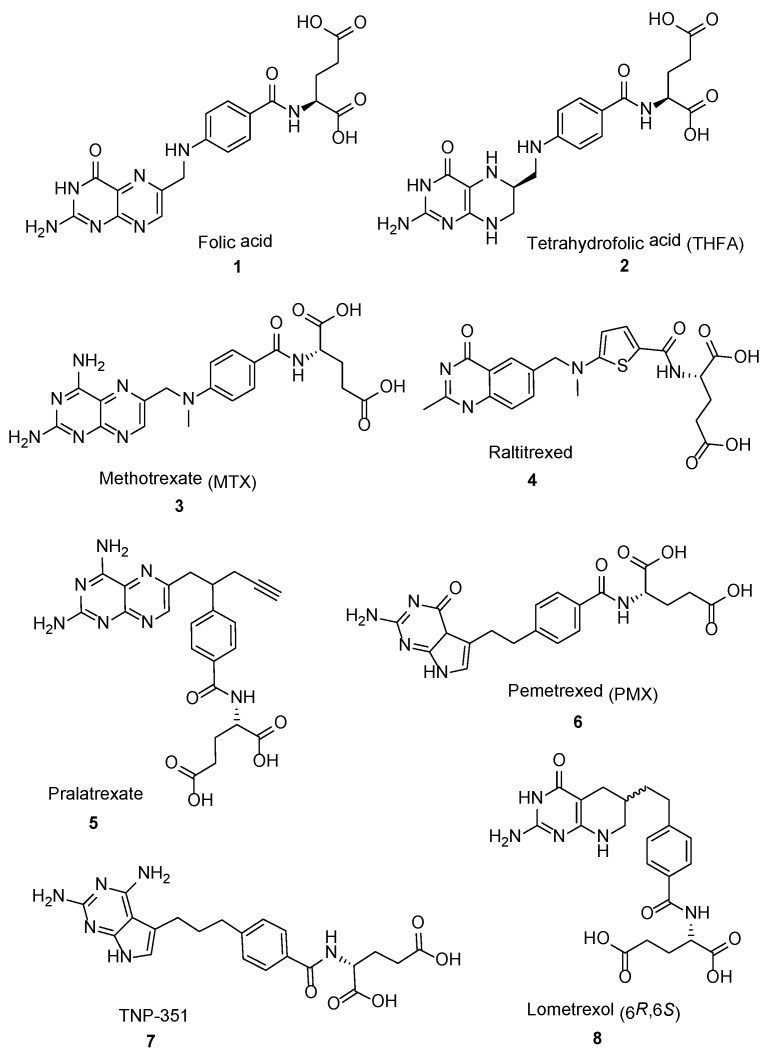
Structure of folic acid (**1**) and tetrahydrofolic acid (THFA) (**2**) and their antimetabolites.

Folic acid (**1**) first has to be reduced to THFA (**2**) by dihydrofolate reductase (Figure 1), after which it can attach various one-carbon groups and transfer them to other molecules. In the reaction, once catalyzed by thymidylate synthase, deoxyuridine monophosphate (deoxy-UMP or dUMP) is converted to deoxythymidine monophosphate (deoxy-TMP or dTMP), producing a methylene group from 5,10-methylene-THFA; the latter is oxidized into dihydrofolic acid and must be reduced again to participate in further reactions. Methotrexate (MTX) and other folic acid antagonists with a high affinity for dihydrofolate reductase (K, 0.01–0.2 nmol/L) disrupt the formation of THFA, causing a deficiency of reduced folates and an accumulation of toxic dihydrofolic acid polyglutamates. At the same time, the transfer reactions of one-carbon groups, which are necessary for the synthesis of purines and dTMP, are inhibited; as a result, the synthesis of nucleic acids and other metabolic processes are disrupted. The toxic action of methotrexate is prevented by calcium folinate (the calcium salt of 5-formyl-THFA), which enters the cell via a reduced folate transporter and is converted into the other THFA derivatives [14] (Scheme 1).

Once it became clear that methotrexate directly inhibits not only dihydrofolate reductase but also the enzymes for the synthesis of purines and thymidylate synthase, the coenzymes of which are reduced folates, a search commenced for folic acid antagonists that selectively inhibit these enzymes. By replacing the N-5, N-8 and N-10 atoms and modifying the side chains of the methotrexate molecule, it was possible to synthesize drugs that retain their inherent ability to form stable polyglutamates inside the cell, but better penetrate the tumor [15], such as the following: raltitrexed, a thymidylate synthase inhibitor; lometrexol, a purine synthesis inhibitor; and pemetrexed, which combines both mechanisms of action [16].

Most folic acid antimetabolites are only partially selective for tumor cells and affect rapidly proliferating normal cells, including bone marrow and gastrointestinal mucosa. Folic acid antagonists act in the S-period and are most active against cells in the logarithmic growth phase [17].

### 2.2. Methotrexate: (S)-2-(4-(((2,4-Diaminopteridin-6-yl)Methyl)(Methyl)Amino)benzamido) Pentanedioic Acid (MTX, Rheumatrex, Amethopterin, Abitrexate, Trexall, Methylaminopterin, Mexate, Metatrexan)

The discovery of the first folic acid antagonist, methotrexate (MTX), with its promising activity for the treatment of a variety of human cancers, prompted the search for other folate analogs [18]. As a structural analogue of folic acid, methotrexate inhibits the activity of the enzyme folate reductase, which prevents the conversion of folic acid into tetrahydrofolic acid, which is involved in cell metabolism and reproduction. Methotrexate is recommended for acute childhood leukemia; chorionepithelioma of the uterus; cancer of the breast, lungs, testicles, and other malignant tumors in adults (in combination with other antiblastoma drugs); and is also used as an immunosuppressive agent.

**Scheme 2:**
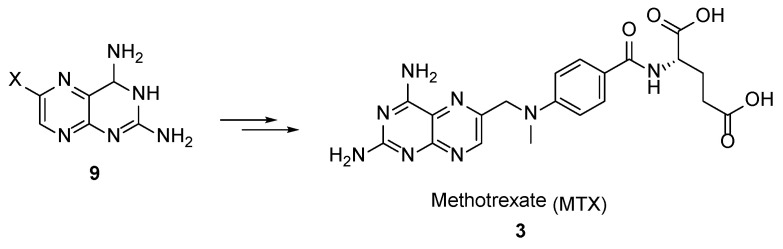
Synthetic strategy towards MTX by the post-modification of 3,4-dihydropteridine-2,4-diamines **9**.

The most common synthetic strategy for the preparation of MTX **3** involves the post-modification of 3,4-dihydropteridine-2,4-diamines **9,** as depicted in Scheme 2. 

**Scheme 3:**
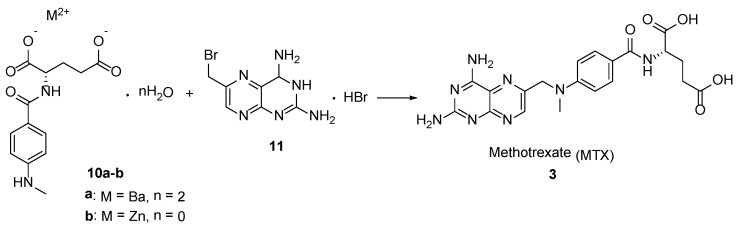
Synthesis of MTX by the reaction between metal salt of *p*-(*N*-methyl)-aminobenzoyl-*L*-glutamic acid and 2,4-diamino-6-bromomethylpteridine hydrobromide.

Thus, MTX was obtained by the reaction of 2,4-diamino-6-bromomethylpteridine hydrobromide **11** with barium salt dehydrate [19] or Zn^2+^ salt [20] of *p*-(*N*-methyl)-aminobenzoyl-*L*-glutamic acid **10** in 87.5% and 56.1% yields, accordingly (Scheme 3).

**Scheme 4:**
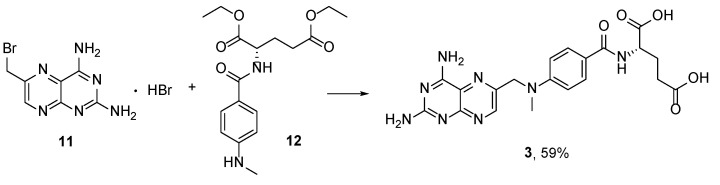
Synthesis of MTX by the reaction of 2,4-diamino-6-bromomethylpteridine hydrobromide **11** with diethyl *p*-(*N*-methyl)-aminobenzoyl-*L*-glutamate **12**.

The reaction of **11** with the diethyl *p*-(*N*-methyl)-aminobenzoyl-*L*-glutamate **12** followed by basic saponification (Scheme 4) provided lower yields of the target product [21].

**Scheme 5:**
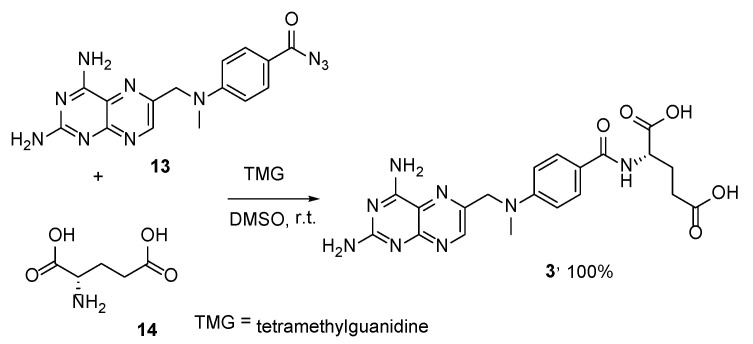
Quantitative synthesis of MTX by the reaction of 4-(*N*-methyl-*N′*-(6″-aminopteroil-methyleno)aminobenzoic acid derivative **13** with L-glutamic acid **14**.

Another approach involves the substitution of the azide group in 4-(*N*-methyl-*N′*-(6″-aminopteroil-methyleno)aminobenzoic acid derivative **13** in a reaction with L-glutamic acid **14** in DMSO at room temperature in the presence of tetramethylguanidine (TMG) as the base (Scheme 5) [22]. The reaction resulted in the corresponding desired MTX in a quantitative yield, which is the main advantage of this method. 

**Scheme 6:**
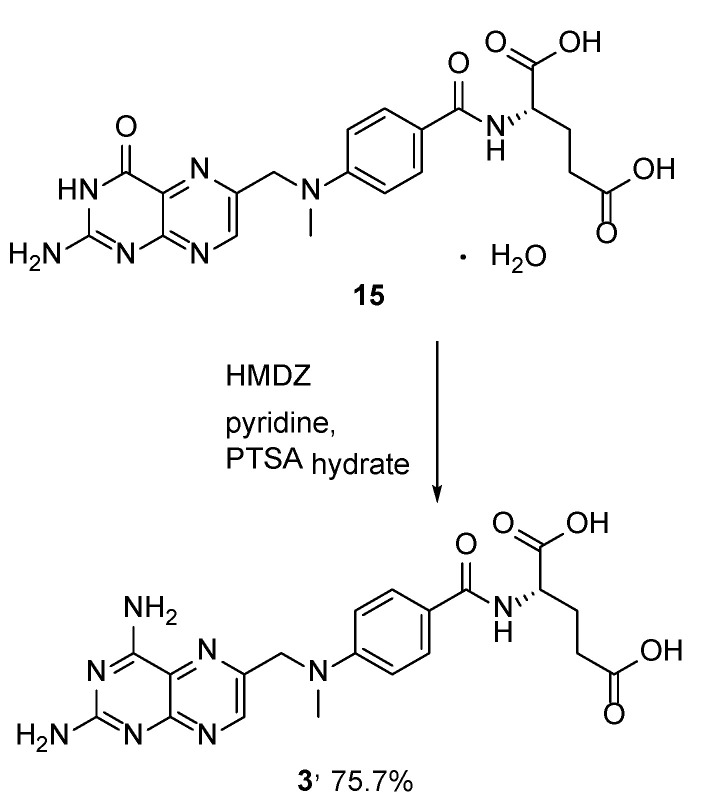
Synthesis of MTX from 4-oxoderivative of MTX (methopterin hydrate) (**15**).

In addition, MTX was obtained in a 75.7% yield by means of the transformation of its more stable and synthetically available 4-oxoderivative (methopterin hydrate) (**15**) in the presence of pyridine, *p*-toluenesulfonic acid monohydrate and 1,1,1,3,3,3-hexamethyl-disilazane (HMDZ) (Scheme 6) [23]. 

**Scheme 7:**
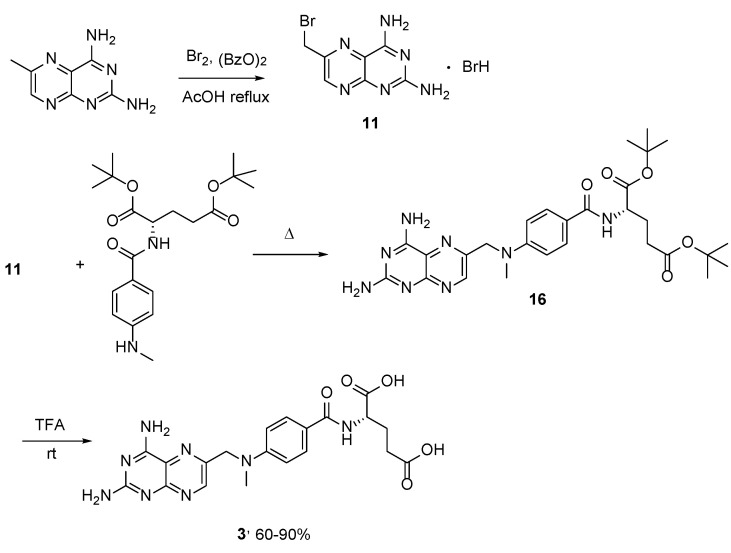
Multistep synthesis of MTX.

Along with MTX, its ^13^C-multilabelled forms with ^13^C-enrichment at 2, 7, 9, 4, 7, 8a, 9 and 2, 4a,b positions were synthesized from the di-*tert*-butyl ester of MTX **16** for the NMR study of the mechanisms of drug–enzyme interactions (Scheme 7) [24]. The reaction was carried out by performing ‘benzylic’ bromination, followed by the substitution of the bromine atom by the di-*t*-butyl *N*-(*p*-methylaminobenzoyl)-*L*-glutamate. The acid treatment of each of the formed methotrexate di-*t*-butyl esters yielded the corresponding ^13^C-enriched methotrexate in 60–90% yields. So far, this is the only method reported for the synthesis of C^13^-MTX.

**Scheme 8:**
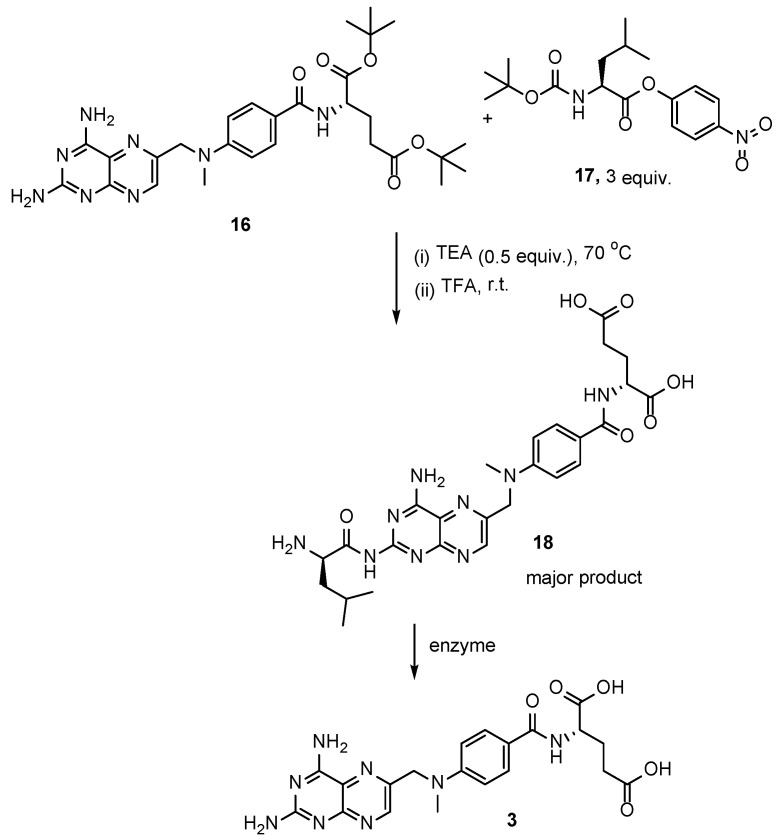
Three-step synthesis of MTX starting from di-*tert*-butyl ester of MTX **16** and *N*-*tert*-butyloxycarbonyl-*L*-leucine derivative **17**.

In another method for the synthesis of MTX **3**, the pro-drug of MTX, *N*-(*L*-α-aminoacyl)-derivative of methotrexate **18**, was initially prepared by a reaction between the di-*tert*-butyl ester of MTX **16** and *N*-*tert*-butyloxycarbonyl-*L*-leucine derivative **17**, followed by the acidic deprotection of protective groups [25]. Subsequently, the obtained pro-drug **18** was successfully converted into MTX via the enzymatic cleavage by porcine microsomal leucine aminopeptidase (Scheme 8). Unfortunately, the authors did not provide any yields due to the format of the publication.

**Scheme 9:**
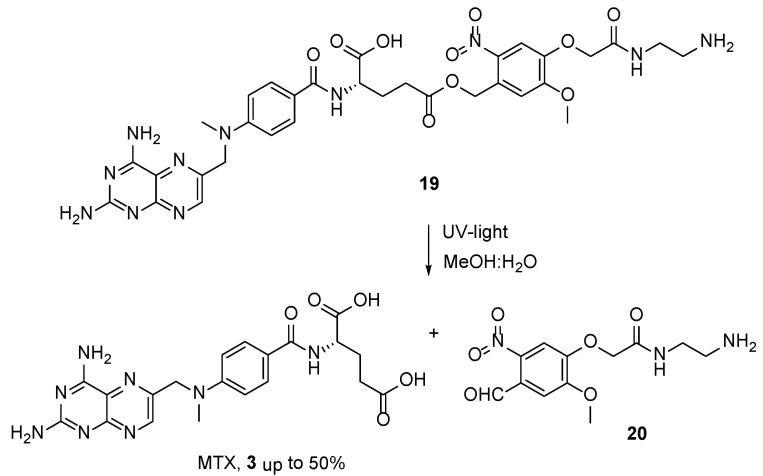
UV-light promoted synthesis of MTX from **19**. Reproduced with the permission of reference [26]. Copyright © 2011, Elsevier Ltd.

Free-form MTX was obtained from the conjugate of the *o*-nitrobenzyl alcohol derivative and MTX **19** during a photolysis experiment in aqueous methanol under UV-light irradiation [26]. This technique was considered by the authors as a possible way to transport the MTX to the cancer cells with the release of MTX free form at up to 50% at a pH level of 7.4 (Scheme 9).

**Scheme 10:**
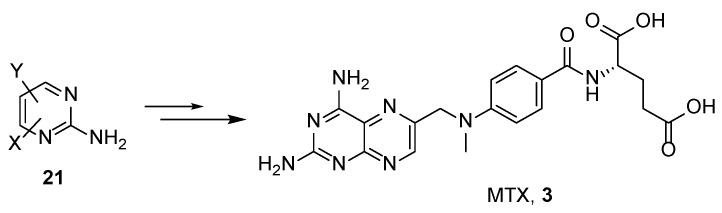
Synthetic strategy towards MTX by the post-modification of aminopyrimidines **21**.

In the literature, there are less common synthetic approaches available that involve the construction of a 3,4-dihydropteridine core starting from aminopyrimidines **21** (Scheme 10).

**Scheme 11:**
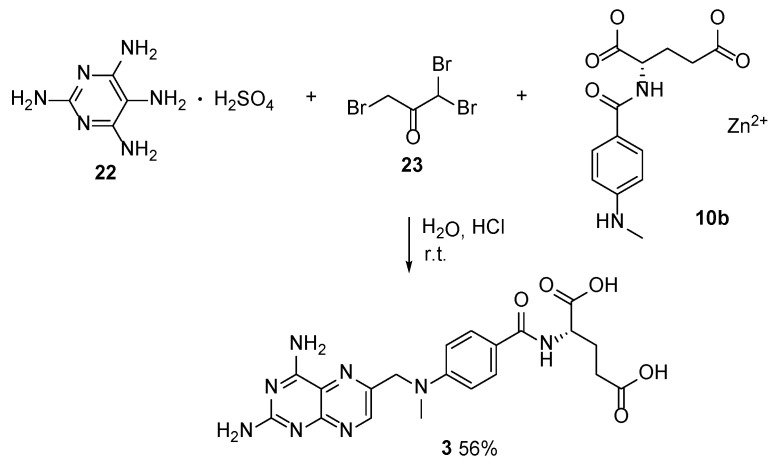
Multicomponent synthesis of MTX under mild reaction conditions.

In this context, MTX was obtained by the tandem multicomponent reaction between Zn^2+^ salt of *N*-(4-*N*-methylaminobenzoil)-*L*-glutamic acid **10b**, 1,1,3-tribromoacetone **23** and 2,2,5,6-tetraaminopyrimidine sulfate **22** under mild conditions (Scheme 11) [27]. This method has a noticeable advantage, such as the possibility to carry out several reactions in one step without the isolation of intermediates during each step. 

**Scheme 12:**
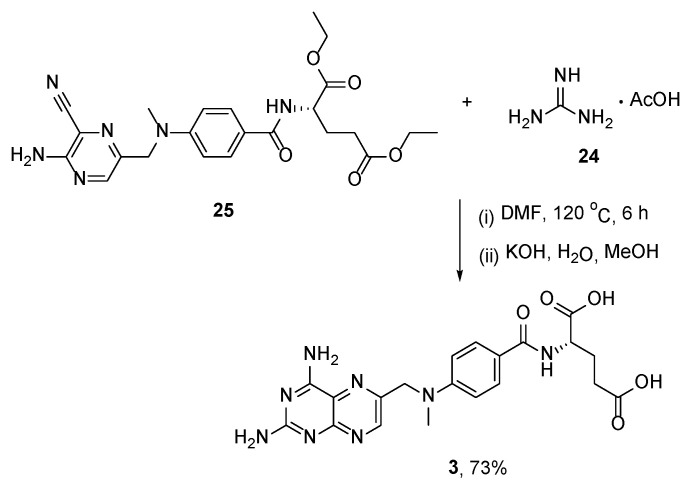
Synthesis of MTX by the reaction between guanidine acetate **24** and diethyl (4-(((5-amino-6-cyanopyrazin-2-yl)methyl)(methyl)amino)benzoyl)glutamate **25**.

In another method, the MTX core was constructed by means of a heterocyclization reaction between commercially available guanidine acetate **24** and easily derived diethyl (4-(((5-amino-6-cyanopyrazin-2-yl)methyl)(methyl)amino)benzoyl)glutamate **25** under heating conditions, followed by basic hydrolysis (Scheme 12) [28].

**Scheme 13:**
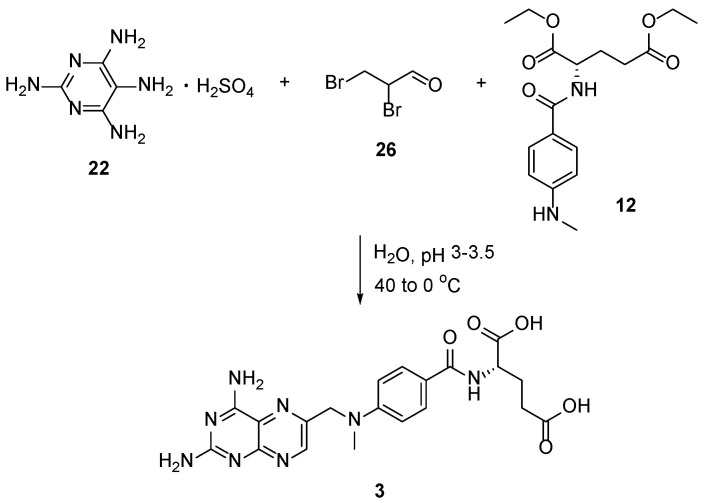
Synthesis of MTX by the reaction between 2,4,5,6-tetraaminopyrimidine hydrosulphate **22**, 2,3-dibromopropionaldehyde **26**, and *N*-4-(methylamino)benzoyl)-*L*-glutamic acid **12** disodium salt under oxidative conditions.

Lastly, the approach for MTX **3** involves a reaction between 2,4,5,6-tetraaminopyrimidine hydrosulphate **22**, 2,3-dibromopropionaldehyde **26**, and *N*-4-(methylamino)benzoyl)-*L*-glutamic acid **12** disodium salt under oxidative conditions (iodine in the presence of KI) (Scheme 13) [29]. In this article, the authors were more concerned about the purity of the obtained compounds than their yields.

### 2.3. Raltitrexed: (2S)-2-[[5-[Methyl-[(2-Methyl-4-oxo-3H-Quinazolin-6-yl)Methyl]Amino] Thiophene-2-Carbonyl]Amino]Pentanedioic Acid (Tomudex, ZD1694)

Raltitrexed (Tomudex) is a more recent, specific, mixed, and non-competitive inhibitor of thymidylate synthase indicated for use in cancer therapy, especially colorectal cancer [30,31,32].

**Scheme 14:**
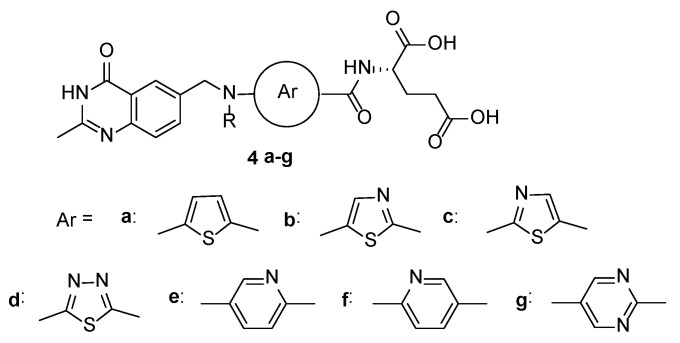
Structures of a series of C2-methyl-N10-alkylquinazoline-based antifolates. Reproduced with the permission of reference [33]. Copyright © 1991, American Chemical Society.

In 1991, Marsham et al. reported the synthesis of a series of C2-methyl-N10-alkylquinazoline-based antifolates, in which the benzene ring was replaced by the heterocycles, i.e., thiophene, thiazole, thiadiazole, pyridine, and pyrimidine (Scheme 14) [33]. 

**Scheme 15:**
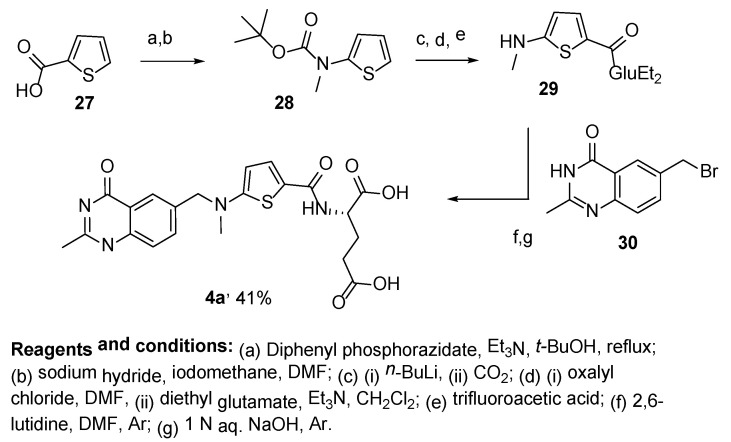
Multistep synthesis of Raltitrexed **4a** starting from thiophene-2-carboxylic acid **27**.

The thiophene system **4a** and its related thiazole **4b** yielded analogues that were considerably more efficient than the parent benzene series as inhibitors of L1210 cell growth. Although, in general, these heterocycles were somewhat poorer inhibitors of the isolated TS enzyme. Raltitrexed **4a** (R = CH_3_) was synthesized in a 41% yield starting with the thiophene-2-carboxylic acid, as shown in Scheme 15.

**Scheme 16:**
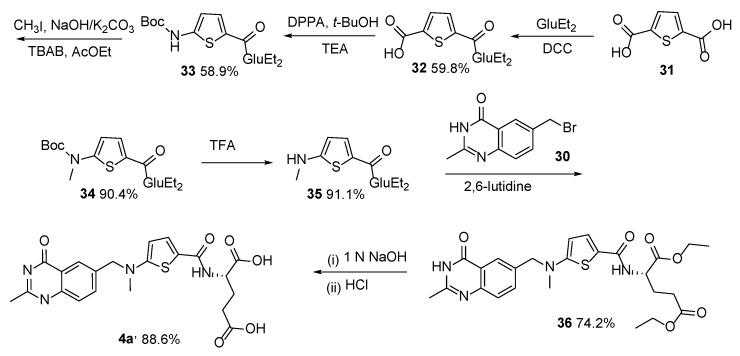
Multistep synthesis of Raltitrexed starting from thiophene-2,5-dicarboxylic acid **31**.

Another route to Raltitrexed was reported that started with thiophene-2,5-dicarboxylic acid **31**, which was then converted in four steps to diethyl (5-(methylamino)thiophene-2-carbonyl)-*L*-glutamate **35**. This was followed by an alkylation reaction of the last one with 6-bromomethyl-2-methyl-4-quinazolinone **30** and basic hydrolysis, which resulted in the target product **4a** (Scheme 16) [34].

**Scheme 17:**
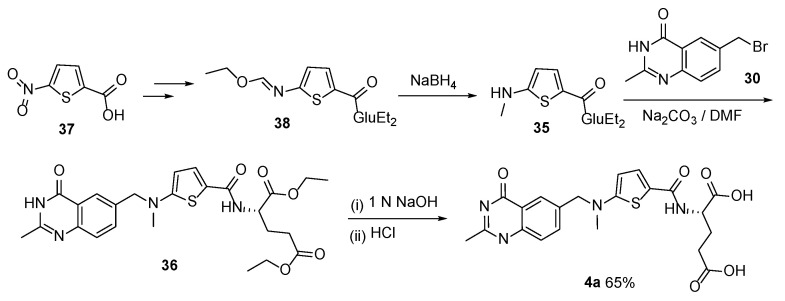
Multistep synthesis of Raltitrexed starting from 5-nitrothiophene-2-carboxylic acid **37**.

A similar route to Raltitrexed was reported by Yao et al. starting with 5-nitrothiophene-2-carboxylic acid **37** via the sequence of NaBH_4_ reduction, alkylation, and saponification (Scheme 17) [35]. The target product was isolated in a lower yield. 

**Scheme 18:**
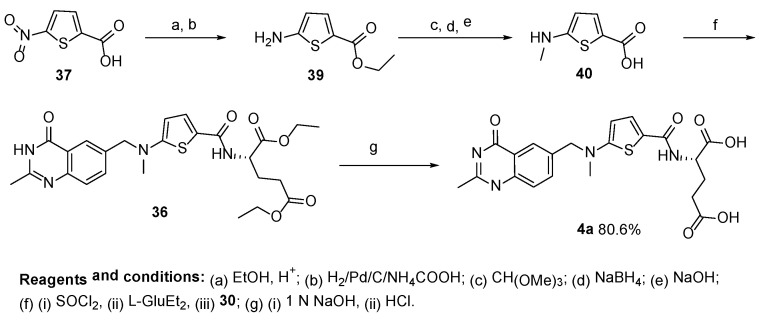
Multistep synthesis of Raltitrexed starting from 5-nitrothiophene-2-carboxylic acid **37**.

Raltitrexed was also prepared by using the same compound in less reaction steps as reported by Xiqun et al. (Scheme 18) [36].

**Scheme 19:**
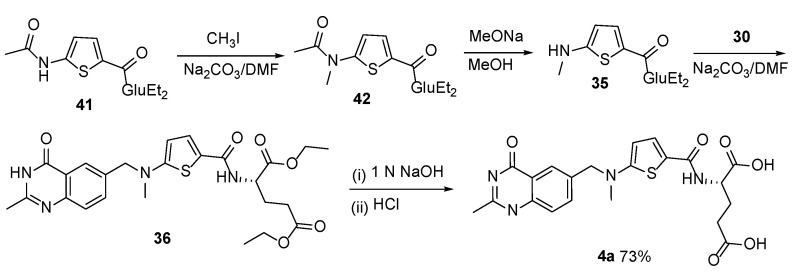
Synthesis of Raltitrexed by diethyl (5-(*N*-methylacetamido)thiophene-2-carbonyl)-*L*-glutamate **41** as the starting material.

Moreover, the most recent and—in our opinion—easiest approach was reported in the work of H. Shaojie et al. regarding Raltitrexed, which involves a four-step sequence using diethyl (5-(*N*-methylacetamido)thiophene-2-carbonyl)-*L*-glutamate **41** as the starting material (Scheme 19) [37].

### 2.4. Pralatrexate (Folotyn): N-4-[1-(2,4-Diaminopteridin-6-yl)Pent-4-yn-2-yl]Benzoyl-L-Glutamic Acid

Pralatrexate **25** is another folate antagonist and antineoplastic agent with confirmed activity for the treatment of relapsed or refractory peripheral T-cell lymphoma (PTCL). Pralatrexate was approved for medical use in the United States in September 2009, as the first treatment for Peripheral T-cell Lymphoma (PTCL) [38,39], an often-aggressive type of non-Hodgkins lymphoma [40].

**Scheme 20:**
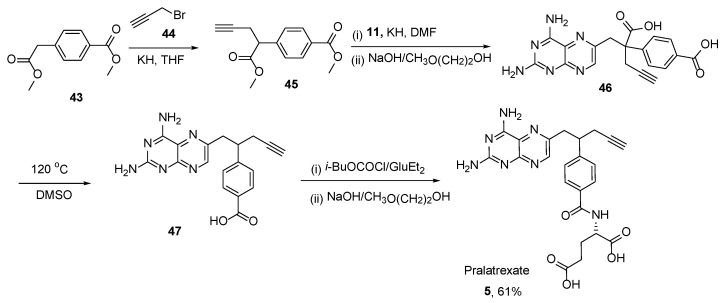
Synthesis of Pralatrexate starting from the reaction between dimethyl homoterephthalate **43** and propargyl bromide. Reproduced with the permission of reference [41]. Copyright © 1993, American Chemical Society.

Successive alkylation of dimethyl homoterephthalate **43** with propargyl bromide **44** and 2,4-diamino-6-(bromomethy1)pteridine **11** followed by ester saponification at room temperature resulted in 2,4-diamino-4-deoxy-10-carboxy-10-propargy1-10-deazapteroic acid **46**. Subsequently, compound **46** was readily decarboxylated by heating in DMSO at 120 °C to yield diamino-10-propargyl-10-deazapteroic acid **47** as a precursor of Pralatrexate **5**. Additionally, the coupling of **47** with diethyl *L*-glutamate followed by ester hydrolysis, yielded Pralatrexate **5** (Scheme 20) [41].

After the abovementioned publication, many improved procedures were reported for the preparation of Pralatrexate [42,43,44,45,46,47,48]. The synthesis of optically pure diastereomers of Pralatrexate has also been reported [49]. 

**Scheme 21:**
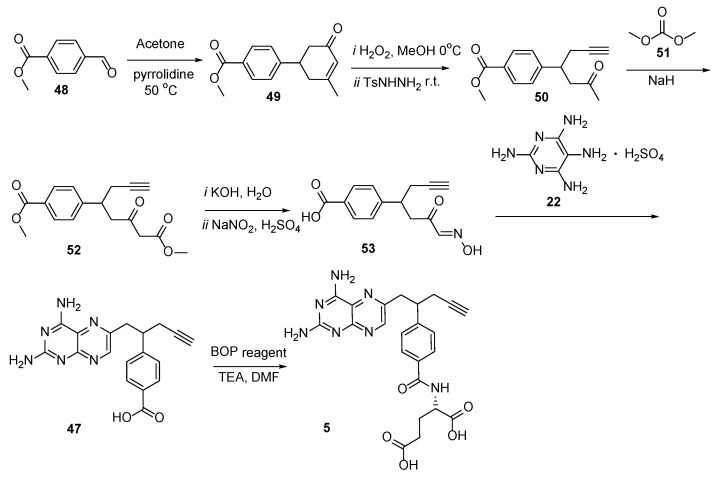
Multistep synthesis of Pralatrexate starting from ethyl 4-formylbenzoate **48**.

Another approach to producing Pralatrexate was developed by Alla et al. (2013), starting with ethyl 4-formylbenzoate **48**; however, the yield was not specified (Scheme 21) [50].

### 2.5. Pemetrexed: (S)-2-(4-(2-(2-Amino-4-oxo-4,7-Dihydro-1H-Pyrrolo[2,3-d]Pyrimidin-5-yl)Ethyl)Benzamido)Pentanedioic Acid (PMX, ALIMTA, LY231514, MTA)

Pemetrexed (PMX) **30** is a folate antagonist and antineoplastic agent, used in the treatment of non-small cell lung cancer [51,52,53,54] and malignant mesothelioma [55]. The mechanism of action of PMX is based on the inhibition of three enzymes responsible for the purine and pyrimidine synthesis—thymidylate synthase (TS), dihydrofolate reductase (DHFR), and glycinamide ribonucleotide formyltransferase [56]—which prevents the formation of DNA and RNA, which are responsible for the growth of normal and cancer cells.

**Scheme 22:**
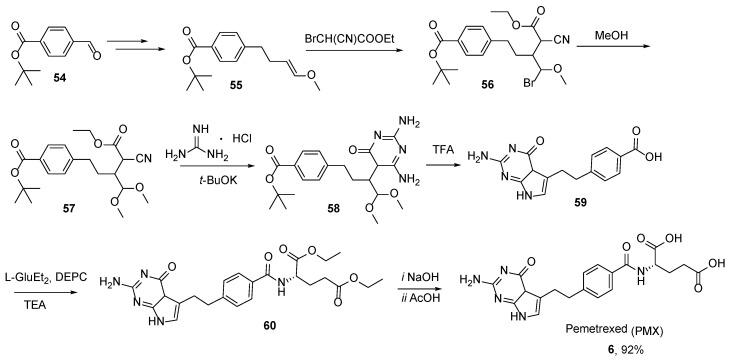
Multistep synthesis of Pemetrexed starting from *tert*-butyl-4-formylbenzoate **54**.

The first synthetic approach toward PMX was reported starting with *tert*-butyl-4-formylbenzoate **54**. This aliphatic precursor was heterocyclized to the PMX diethyl ester **60** in a few steps, which was converted to PMX **6** by performing hydrolysis (Scheme 22) [57].

**Scheme 23:**
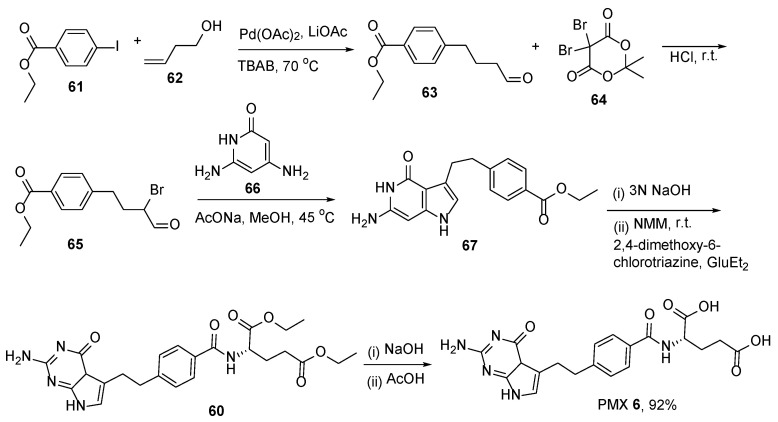
Synthesis of Pemetrexed starting from ethyl 4-iodobenzoate **61**, and 1-butene-4-ol **62** through Heck cross-coupling reaction. Reproduced with the permission of reference [58]. Copyright © 2013, American Chemical Society.

Mitchell-Ryan et al. reported the synthesis of 5-substituted pyrrolo[2,3-*d*]pyrimidine antifolates with one-to-six bridge carbons and a benzoyl ring in the side chain as antitumor agents [58]. The compound with a 4-carbon bridge was the most active analogue and it potentially inhibited the proliferation of the folate receptor (FR) α-expressing Chinese hamster ovary and KB human tumor cells. PMX was synthesized from ethyl 4-iodobenzoate **61**, and 1-butene-4-ol **62** using a Heck cross-coupling reaction followed by bromination of the aldehyde at alpha-position. Further heterocyclization with basic hydrolysis and the formation of amide from diethyl-*L-*glutamate resulted in acid derivative **60**. In the final step, PMX **6** was obtained by the basic hydrolysis of the ester groups in a glutamate moiety (Scheme 23).

As an improvement to the abovementioned method, the preparation of lysin salt of PMX was reported [59].

Michalak et al. reported the synthesis of PMX along with its common impurities/side products, starting with 4-[2-(2-amino-4-oxo-4,7-dihydro-1*H*-pyrrolo[2,3-*d*]pyrimidin-5-yl)ethyl]benzoic acid **28** [60].

**Scheme 24:**
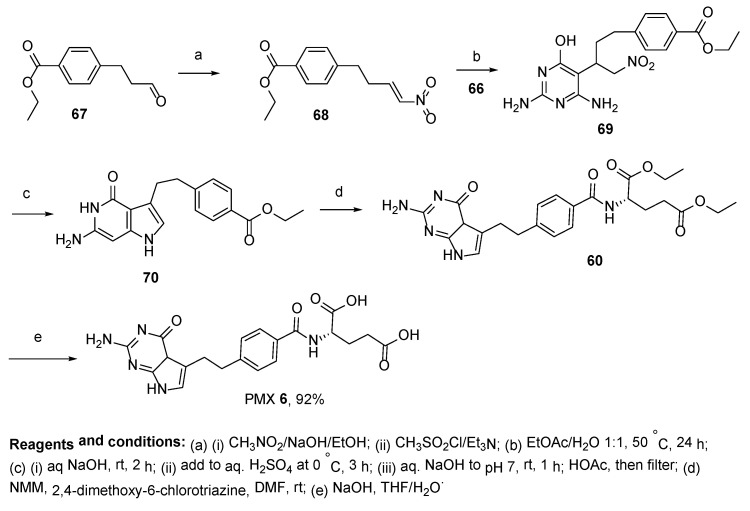
Synthesis of Pemetrexed starting from ethyl-4-(3-oxopropyl)benzoate **67**.

In the method reported by Tailor et al. for the synthesis of PMX, ethyl-4-(3-oxopropyl)benzoate **67** was used as a starting compound [61,62,63]. After the Henry reaction with nitromethane, the product was converted to the semi-product with 2,6-diaminopyrimidin-4-ol **69**. The heterocyclization of this semi-product resulted in pyrrolo[2,3-*d*]pyrimidine derivative **70**, which, followed by its functionalization with diethyl-*L*-glutamate and basic hydrolysis, resulted in the desired product **6** in a 92% yield (Scheme 24).

**Scheme 25:**
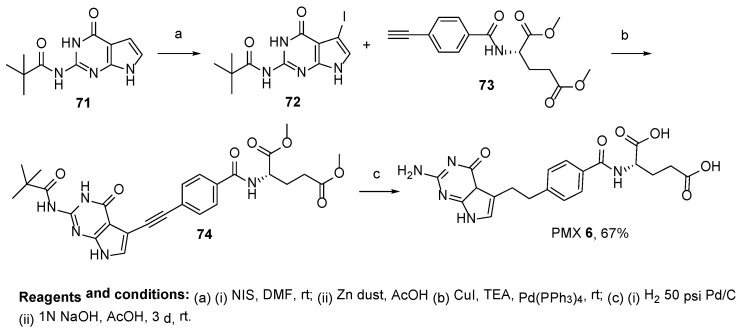
Synthesis of Pemetrexed starting from dimethyl (4-ethynylbenzoyl)-*L*-glutamate **73** and *N*-(4-oxo-4,7-dihydro-3*H*-pyrrolo[2,3-*d*]pyrimidin-2-yl)pivalamide **71**.

The same research group reported an improved synthesis of PMX, starting from dimethyl (4-ethynylbenzoyl)-*L*-glutamate **73** and *N*-(4-oxo-4,7-dihydro-3*H*-pyrrolo[2,3-*d*]pyrimidin-2-yl)pivalamide **71** [64]. The sequence of iodination, reduction, Sonogashira cross-coupling, reduction reactions, and basic hydrolysis in the last step, resulted in the final product, PMX, in a 67% yield (Scheme 25).

**Scheme 26:**
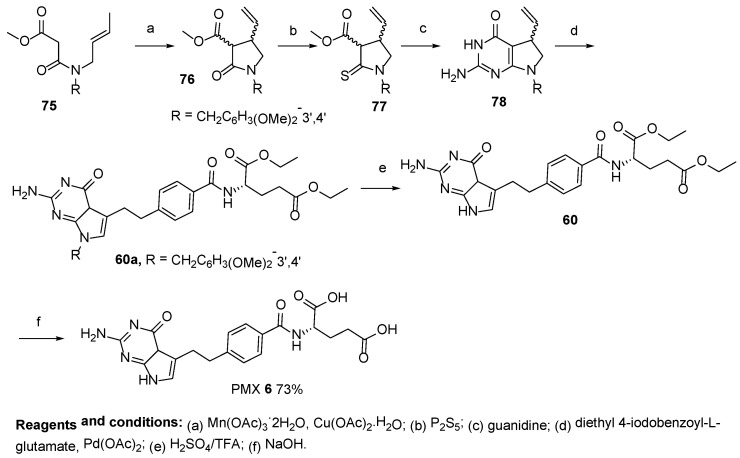
Synthesis of Pemetrexed starting from methyl (*E*)-3-(but-2-en-1-yl(3,4-dimethoxybenzyl)amino)-3-oxopropanoate **75**.

The same authors also reported the synthesis of PMX starting from methyl (*E*)-3-(but-2-en-1-yl(3,4-dimethoxybenzyl)amino)-3-oxopropanoate [65]. The last one was cyclized to methyl-1-(3,4-dimethoxybenzyl)-2-oxo-4-vinylpyrrolidine-3-carboxylate by the reaction of Mn(III) and Cu(II) acetates. The oxo-group was then converted to the thioxo-group upon treatment with P_2_S_5_. After the heterocyclization reaction, the obtained 2-amino-7-(3,4-dimethoxybenzyl)-5-vinyl-4*a*,5,6,7-tetrahydro-4*H*-pyrrolo[2,3-*d*]pyrimidin-4-one was subjected to a Heck cross-coupling reaction with diethyl 4-iodobenzoylglutamate. Additionally, the coupling product was identified as one with unexpected double bond migration products in vinyl-bridged pyrrolinopyrimidine to form the ethano-bridged pyrrolopyrimidine. Thus, the authors avoided the reduction of the unsaturated bridge and the subsequent oxidation of the pyrroline ring at the same time. According to the authors, the protection of the N-7 position eliminates the PMX cell growth’s inhibitory activity. In addition, deprotection of the N-7 position was finally achieved upon treatment with a H_2_SO_4_/TFA mixture to facilitate the PMX precursor in a 30% yield, which resulted in the target product after saponification (Scheme 26).

**Scheme 27:**
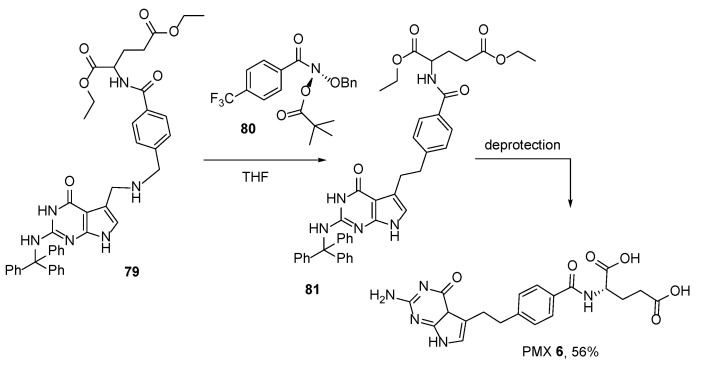
Synthesis of Pemetrexed by the reaction of anomeric amide agent with secondary amine precursor followed by the deprotection of protective groups.

Finally, very recently, a method for PMX synthesis was developed by means of the reaction of an anomeric amide agent with a secondary amine precursor followed by the deprotection of protective groups (Scheme 27) [66].

### 2.6. TNP-351: (2S)-2-[[4-[3-(2,4-Diamino-7H-Pyrrolo[2,3-d]Pyrimidin-5-yl)Propyl]benzoyl]Amino]Pentanedioic Acid (HY-19095)

TNP-351 is another antifolate from the same family as PMX. As a dihydrofolate reductase (DHFR) inhibitor, TNP-351 has good potential for the treatment of not only leukemia cells but also solid tumor cells, both in vitro and in vivo [67]. The structure of TNP-351 contains three methylene bridges instead of two as in PMX and two amino groups in pyrimidine core.

**Scheme 28:**
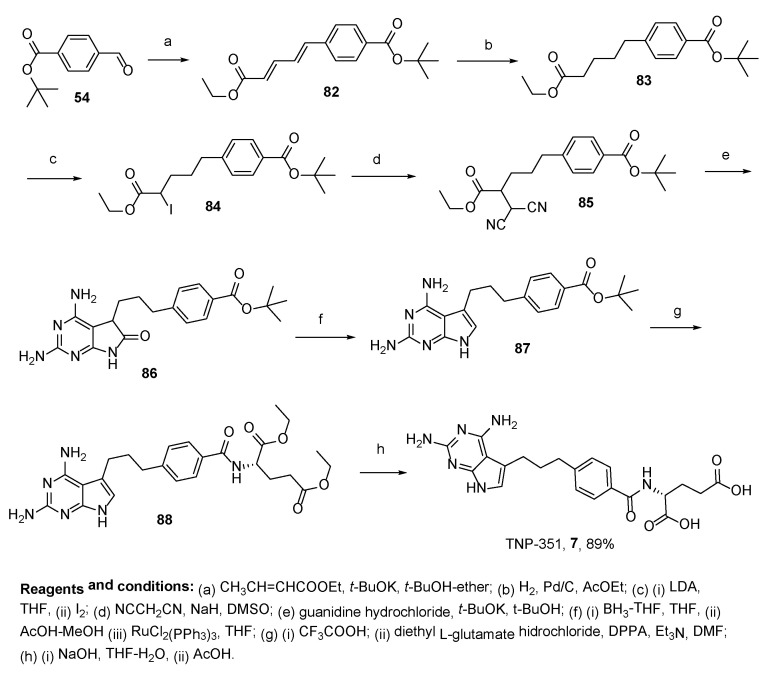
Multistep synthesis of TNP-351.

So far, only two synthetic approaches to TNP-351 **7** have been reported; the first one includes construction of the key intermediary acyclic skeleton, 5-[4-(*tert*-butoxycarbonyl)phenyl]-2-(dicyanomethyl)pentanoate **85**, cyclization with guanidine, followed by reduction to pyrrolo[2,3-*d*]pyrimidine derivatives **87**, and subsequent glutamate coupling and saponification. These antifolates were more growth-inhibitory by approximately one order of magnitude than methotrexate (MTX) against KB human epidermoid carcinoma cells and A549 human non-small cell lung carcinoma cells with in vitro culture (Scheme 28) [68].

**Scheme 29:**
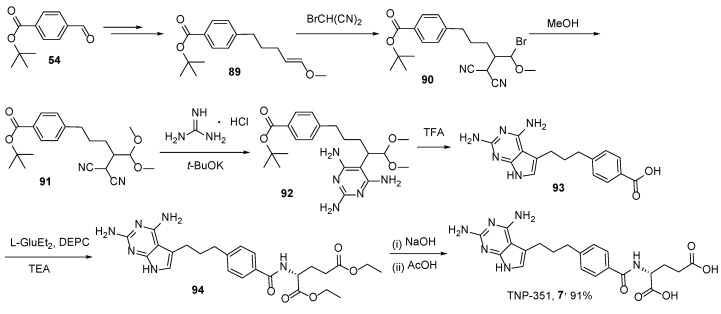
Multistep synthesis of TNP-351.

The second method belongs to the same article, where the synthesis of TNP-351 has been reported along with PMX synthesis (Scheme 29) [57].

### 2.7. Lometrexol: (2S)-2-[[4-[2-[(6R)-2-Amino-4-oxo-5,6,7,8-Tetrahydro-1H-Pyrido[2,3-d]Pyrimidin-6-yl]Ethyl]Benzoyl]Amino]Pentanedioic Acid (LY 264618, DDATHF-B, Lometrexolum)

Lometrexol (6*R*)-**8** is a folate analogue antimetabolite with antineoplastic activity [69,70,71]. As the 6*R* diastereomer of 5,10-dideazatetrahydrofolate, lometrexol inhibits glycinamide ribonucleotide formyltransferase (GARFT), the enzyme that catalyzes the first step in the de novo purine biosynthetic pathway, thereby inhibiting DNA synthesis, arresting cells in the S phase of the cell cycle, and inhibiting tumor cell proliferation. The agent is active against tumors that are resistant to the folate antagonist methotrexate.

Lometrexol has been used in trials for the treatment of lung cancer, drug/agent toxicity by tissues/organs, as well as for the treatment of unspecified adult solid tumors.

**Scheme 30:**
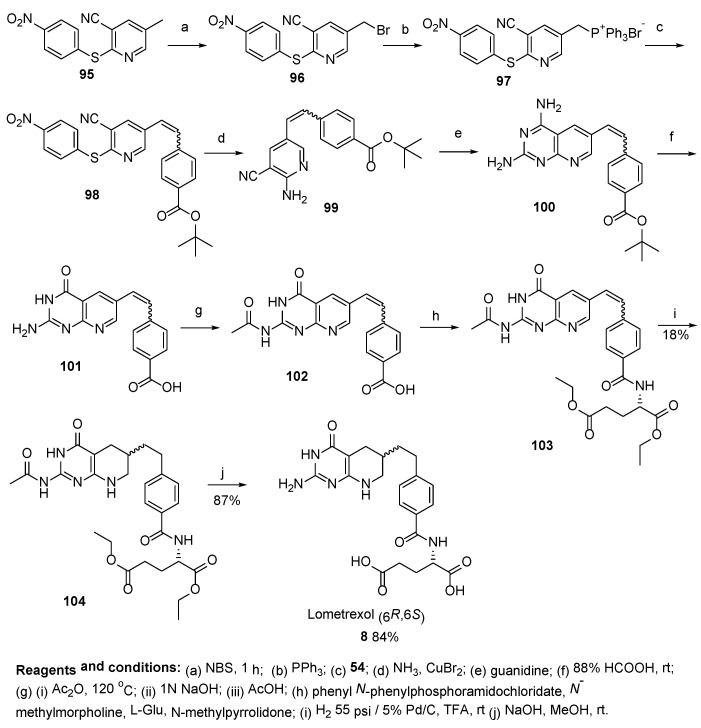
Synthesis of Lometrexol starting from 5-methyl-2-((4-nitrophenyl)thio)nicotinonitrile **95**.

Taylor et al. reported several approaches to Lometrexol. The first of their approaches relates to the synthesis of (mixture of diastereomers) *(6S,6R)*-Lometrexol **8** with a satisfactory yield starting from 5-methyl-2-((4-nitrophenyl)thio)nicotinonitrile **95** (Scheme 30) [72].

**Scheme 31:**
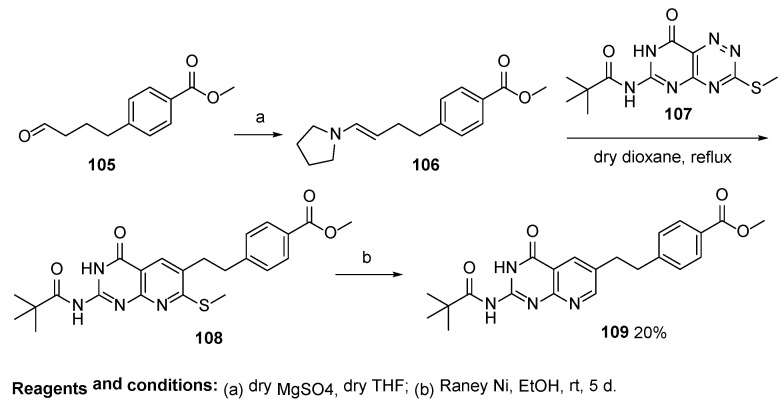
Synthesis of key intermediate **109**.

A key intermediate **109** for the subsequent synthesis of *(6S,6R)*-Lometrexol was also prepared by Taylor et al. via a regiospecific intermolecular inverse electron demand Diels-Alder reaction between fused 1,2,4-triazines, 2-*N*-pivaloyl-7-substituted-6-azapterins, and enamine (Scheme 31) [73].

**Scheme 32:**
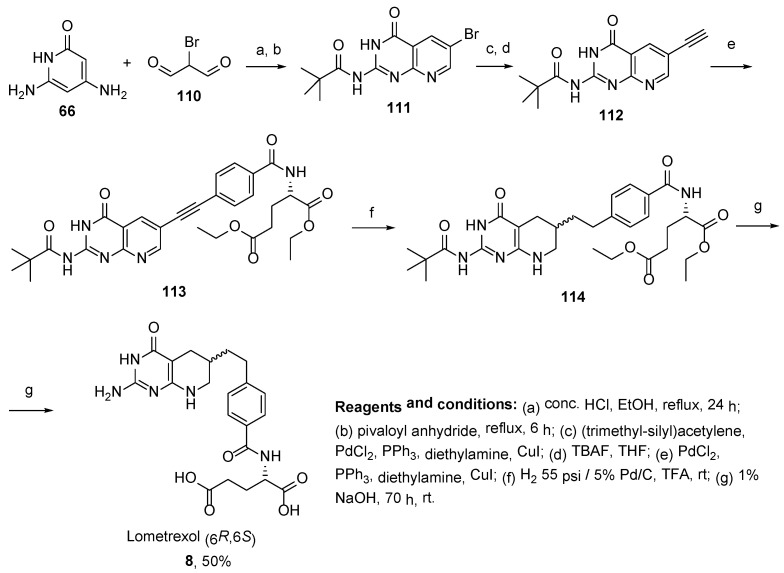
Synthesis of *(6S,6R)*-Lometrexol **8** via *N*-(6-bromo-4-oxo-3,4-dihydropyrido[2,3-*d*]pyrimidin-2-yl)pivalamide starting from 2,6-diaminopyrimidin-4(3*H*)-one.

In another work, Taylor et al. developed a convenient method for the synthesis of *(6S,6R)-*Lometrexol **8** with good yield via *N*-(6-bromo-4-oxo-3,4-dihydropyrido[2,3-*d*]pyrimidin-2-yl)pivalamide starting with 2,6-diaminopyrimidin-4(3*H*)-one (Scheme 32) [74].

**Scheme 33:**
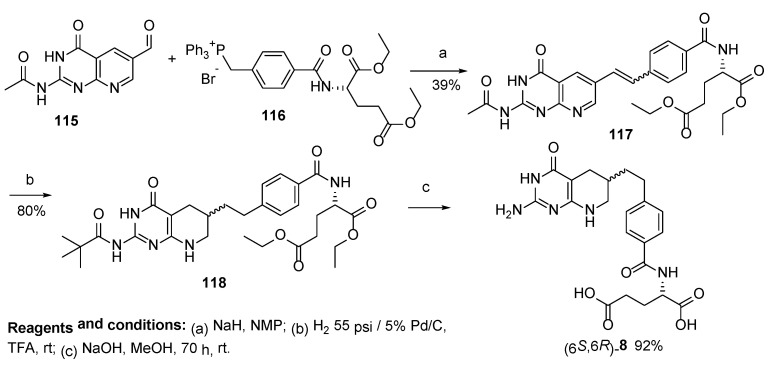
Synthesis of *(6S,6R)*-Lometrexol **8** by the Wittig olefination of 2-acetyl-6-formyl-5-deazapterine.

Boschelli et al. performed Wittig olefination of 2-acetyl-6-formyl-5-deazapterine to prepare *(6S,6R)*-Lometrexol in three synthetic steps instead of Sonogashira cross-coupling of 2-pyvaloyl-6-formyl-5-deazapterine (Scheme 33) [75].

**Scheme 34:**
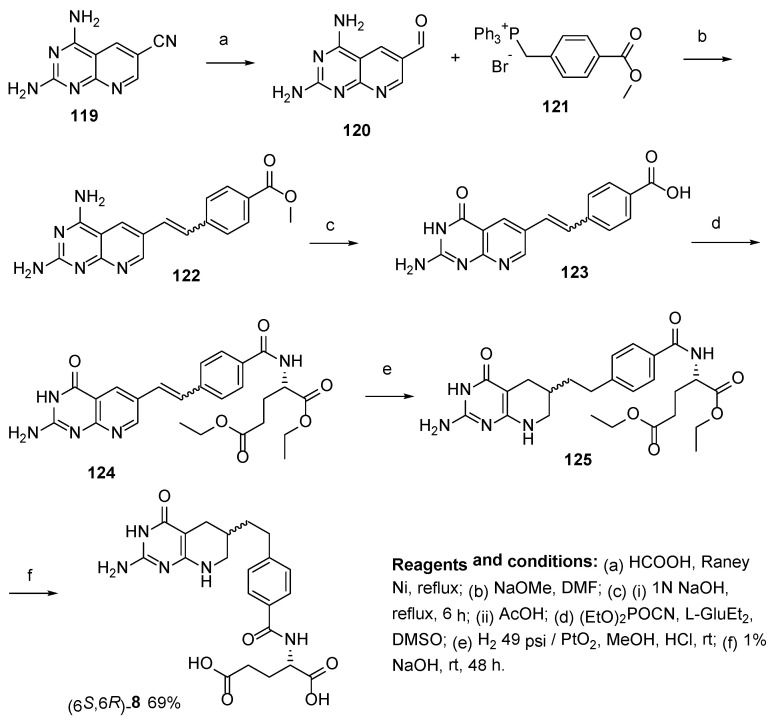
Synthesis of (6*S*,6*R*)-Lometrexol starting from 2,4-diaminopyrido[2,3-*d*]pyrimidine-6-carboxaldehyde.

Similarly, Wittig olefination was used by Piper et al. for the synthesis of (6*S*,6*R*)-Lometrexol starting from 2,4-diaminopyrido[2,3-*d*]pyrimidine-6-carboxaldehyde **120**, derived from 6-carbonytrile, and [4-(methoxycarbonyl)benzylidene]triphenylphosphorane to yield 9,10-ethenyl precursor **122** [76]. Standard hydrolytic deamination produced 5,10-dideazafolic acid **123,** which was further converted to 5,10-dideazaaminopterin via a coupling reaction with dimethyl *L*-glutamate by using (EtO)_2_POCN, followed by hydrogenation and ester hydrolysis which led to the final product **8** (Scheme 34).

**Scheme 35:**
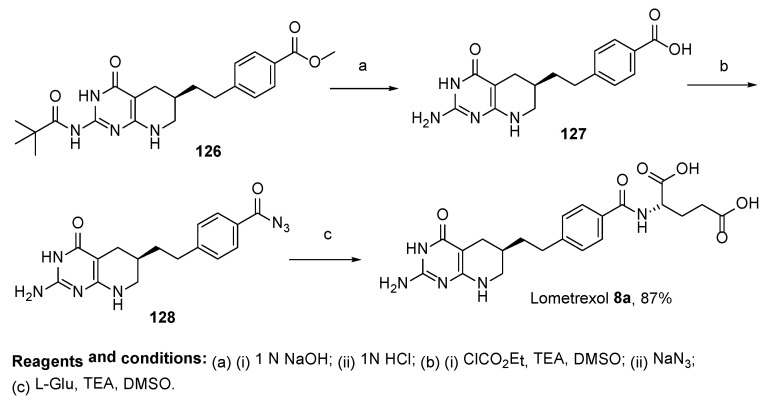
Synthesis of 6*R*-Lometrexol starting from a double deprotected DDAH_4_Pte–OH **126**.

Currently, only two synthetic approaches toward diastereometrically pure 6*R*-Lometrexol are reported in the literature. In this context, the synthesis of 6*R*-Lometrexol was carried out starting from a double deprotected DDAH_4_Pte–OH **126**, which was obtained with preparative chiral-HPLC in a mixture of diastereomers derived from the route based on the work of Taylor et al. [77]. After the transformation of the benzoic acid residue to a derivative which includes azides, the azide derivative was converted to the final product by the reaction of *L*-glutamic acid in DMSO in the presence of TEA (Scheme 35) [78].

**Scheme 36:**
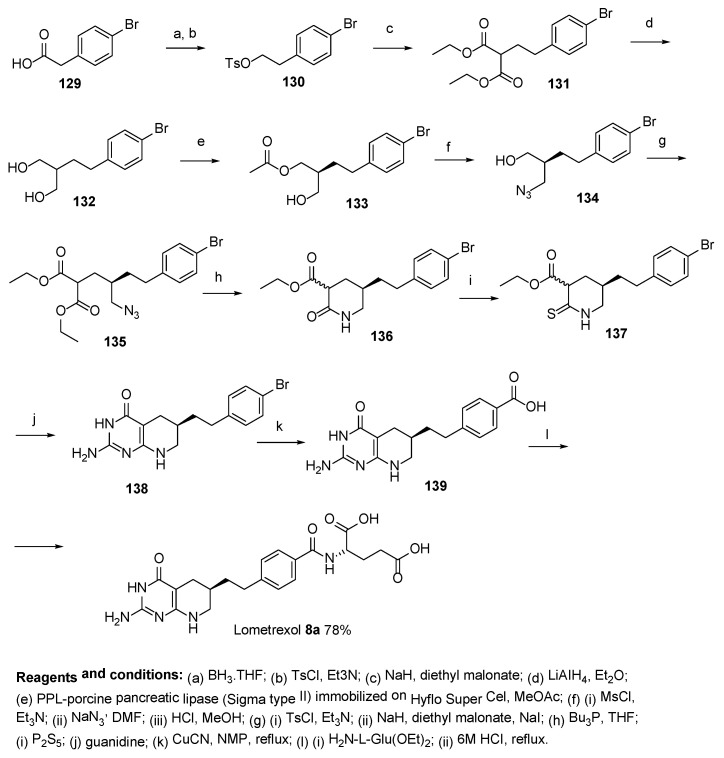
Multistep synthesis of Lometrexol starting from 2-(4-bromphenyl)acetic acid. Reproduced with the permission of references [79]. Copyright © 1989, Elsevier Ltd.

In another approach, the lipase-catalyzed enantioselective esterification of 2-(4-bromophenethyl)propane-1,3-diol, derived in several steps from 2-(4-bromphenyl)acetic acid, was utilized in the asymmetric synthesis of key (*R*)-2-amino-6-(4-bromophenethyl)-5,6,7,8-tetrahydropyrido[2,3-*d*]pyrimidin-4(3*H*)-one, which resulted in the target product in two synthetic steps (Scheme 36) [79,80].

## 3. Conclusions and Future Perspectives

In summary, this review represents the analysis of the most up-to-date synthetic approaches for the synthesis of therapeutically significant analogues of folic acid, such as Lometrexol, Methotrexate, Pemetrexed, Pralatrexate, Raltitrexed, and TNP-351. Among the other folic acid analogues exhibiting antimalarial/antiprotozoal [81] and broad-spectrum antimicrobial activity [82,83,84], the importance and effectiveness of the abovementioned six analogues of folic acid as drugs or drug candidates for the treatment of diseases with a social significance, such as various types of cancers, severe psoriasis, and rheumatoid arthritis, were reported in a large number of original research publications and review articles [18,30,31,32,40,51,52,53,54,55,67,69,70,71]. Even though folates were reported to be somehow connected with the severeness of COVID-19 [85,86,87], several recent studies suggested the effectiveness of antifolates for the therapy of patients with coronavirus SARS-CoV-2 [85,88,89], along with the enhancement of the antiviral efficacy of remdesivir [88], treatment of fungal infections with COVID-19-like symptoms [90], as well as treatment of fungal infections among COVID-19 patients [90].

Most of the synthetic strategies for these important scaffolds presented in research articles and patents are based on similar approaches and have only minor differences from each other. So far, no attention has been paid to methods based on transitional metal (TM)-catalyzation or the TM-free direct C-H-activation/C-H-functionalization of aza-aromatic rings as the most atom- and step-efficient approaches. We hope that our review will encourage future interest in this research area.

## Data Availability

Not applicable.
